# Gaseous Microemboli and Postoperative Delirium in Coronary Artery Bypass Grafting

**DOI:** 10.3390/jcm14145123

**Published:** 2025-07-18

**Authors:** Vladimir Tutuš, Milica Paunović, Nina Rajović, Nataša Milić, Miloš Matković, Radmila Karan, Svetozar Putnik, Nemanja Aleksić, Danijela Trifunović Zamaklar, Marko Jugović, Ilija Bilbija, Selena Nešić, Dejan Marković

**Affiliations:** 1 Department of Anesthesia, University Clinical Centre of Serbia, 11000 Belgrade, Serbia; vltutus@yahoo.com (V.T.); karan.radmila@gmail.com (R.K.); marko_j57@yahoo.com (M.J.); selena91bg@gmail.com (S.N.); drdejanmarkovic@gmail.com (D.M.); 2 Faculty of Medicine, University of Belgrade, 11000 Belgrade, Serbia; dr.matko@hotmail.com (M.M.); svetozar073@yahoo.com (S.P.); ner.vuk@hotmail.com (N.A.); danijelatrif@gmail.com (D.T.Z.); i.bilbija@yahoo.com (I.B.); 3 Institute for Medical Statistics and Informatics, Faculty of Medicine, University of Belgrade, 11000 Belgrade, Serbia; nina94rajovic@gmail.com (N.R.); silly_stat@yahoo.com (N.M.); 4 Department of Cardiac Surgery, University Clinical Centre of Serbia, 11000 Belgrade, Serbia; 5 Department of Cardiology, University Clinical Centre of Serbia, 11000 Belgrade, Serbia

**Keywords:** CABG, cardiopulmonary bypass, gaseous microemboli, postoperative delirium

## Abstract

**Background**: Postoperative delirium (POD) is a neurocognitive syndrome affecting patients undergoing surgery. It is a frequent complication of coronary artery bypass grafting (CABG) and is associated with higher morbidity, mortality and treatment costs. This study aimed to investigate the relationship between gaseous microemboli (GME) load during cardiopulmonary bypass (CPB) and subsequent POD in patients undergoing CABG. **Methods**: In total, 102 patients undergoing elective on-pump CABG were evaluated in this observational study. An ultrasonic microbubble counter, with probes placed on the arterial and venous lines, was used during CPB to evaluate the GME load for each patient. During the first postoperative week, the patients were examined for the presence of POD. **Results**: Patients diagnosed with POD had higher number of bubbles in the arterial CPB line (5382.8 (4127.8–6637.8) vs. 2389.4 (2033.9–2745.0), *p* < 0.001), higher volume of bubbles in both the venous (24.2 µL (16.8–31.6) vs. 12.4 µL (9.7–15.1), *p* = 0.004) and arterial lines (1.82 µL (1.43–2.21) vs. 0.29 µL (0.22–0.36), *p* < 0.001), lower quality factor (QF) values (*p* = 0.039), a lower venoarterial reduction in bubble number (83.0% (77.8–88.1) vs. 92.4% (90.9–93.8), *p* = 0.001) and a lower venoarterial reduction in bubble volume (88.8% (85.4–92.2) vs. 96.3% (95.2–97.3), *p* < 0.001) compared to the patients without POD. Older age (*p* = 0.005), a lower reduction in bubble volume (*p* < 0.001) and lower QF values (*p* = 0.004) were significant independent predictors of POD. **Conclusions**: Our findings indicate a strong association between GME and the occurrence of POD, which entails that all available actions should be implemented to prevent their generation and facilitate the elimination of GME from the CPB circuit.

## 1. Introduction

Neurological and neuropsychiatric events are the most frequent complications of coronary artery bypass grafting (CABG) [1,2,3,4]. Postoperative delirium (POD) is a neurocognitive syndrome that affects patients who have undergone surgery under general anesthesia. It is associated with higher morbidity and mortality and an increased cost of treatment [5,6].

The incidence of POD is highly variable across populations, ranging from 5 to 50% in patients undergoing CABG. Various preoperative (older age, ASA physical status >2, diabetes mellitus, anemia, cognitive impairment, cerebrovascular and cardiovascular disease), intraoperative (site of surgery—especially cardiac, orthopedic and abdominal surgery—longer duration of surgery and anesthesia, extensive blood loss and transfusion) and postoperative (longer duration of mechanical ventilation, intensive care unit stay, hospital stay) risk factors for POD have been identified [7,8,9]. The exact mechanism of POD in patients undergoing CABG is yet to be elucidated, although the roles of cardiopulmonary bypass (CPB), embolization and prolonged intraoperative arterial hypotension have been postulated [10,11]. Gaseous microemboli (GME) are suspected to be one of the causes of POD in patients undergoing CABG through several mechanisms. Besides simply obstructing blood flow in cerebral capillaries, GME compresses endothelial cells, causing their dysfunction and disruption of the blood–brain barrier, with leakage of fluid into the interstitium. The presence of GME induces an inflammatory response mediated by leukocyte aggregation, the release of proteolytic enzymes and reactive oxygen species, with complement activation, as well as platelet aggregation and coagulation cascade activation, further contributing to tissue damage [12,13,14].

This study aimed to investigate the association between the number and volume of GME during CPB and the occurrence of POD.

## 2. Materials and Methods

This was an observational (cross-sectional) study with prospective data collection. Consecutive patients undergoing isolated CABG at the Department for Cardiac Surgery, University Clinical Center of Serbia from 1 January to 31 July 2024 were screened. Only patients who underwent first-time isolated on-pump CABG were included in the study. Patients with psychiatric diagnoses or cognitive deficits, as well as patients undergoing redo, off-pump, or minimally invasive cardiac surgery were excluded from the study.

Basic sociodemographic characteristics, body composition measurements and the presence of comorbidities were collected from the 102 enrolled patients’ medical histories. A standard battery of preoperative laboratory analyses was obtained for each patient.

General anesthesia was induced using etomidate (0.2 mg/kg IV) and maintained using sevoflurane (0.3–2.0 vol%) and sufentanil (TCI 0.3–1.0 ng/mL) with targeted BiSpectral Index (BIS) values from 40 to 60.

CPB was conducted following a standardized procedure (ACT >480 s, mild hypothermia, nonpulsatile flow of 2.4 L·min^–1^·m^–2^ multiplied by body surface area of patient, mild hemodilution, induction of cardiac arrest by anterograde infusion of crystalloid St. Thomas II cardioplegia during aortic cross-clamp for distal anastomoses). Standardized CPB kits comprising cannulae, tubing, a heat-exchanger, a roller pump, a venous reservoir and one of the three available oxygenators (Terumo Capiox FX25, Terumo Cardiovascular, 6200 Jackson Road, Ann Arbor, MI 48103-9300 USA; Sorin Inspire 8 or Sorin Inspire 8F, LivaNova Inc., 20 Eastbourne Terrace, London, W2 6LG, UK) were used.

Data on the duration of surgery, CPB, aortic cross-clamp (AoCl) and reperfusion, pO2, volume of administered cardioplegia and intraoperative blood salvaged by the cell saver (CS), body temperature and lactate and glucose levels during CPB were collected.

The bubble number (defined as the total number of bubbles counted in time intervals of 1 s) and bubble volume (defined as the volume of all bubbles during CPB) in the CPB circuit proximally and distally to the oxygenator (in the arterial and venous lines) were assessed using the Gampt BC200 (GAMPT mbH, Hallesche Str. 99F, 06217 Merseburg, Germany) acoustic microbubble counter. Ultrasonic probes were placed around the CPB circuit tubing at two defined positions in respect to the oxygenator. From these values, we further extrapolated the number reduction (defined as the percentage of bubble number decrease between the venous and arterial lines), volume reduction (defined as the percentage of bubble volume decrease between the venous and arterial lines), quality factor (QF) (defined as the percentage of the total number of bubbles detected by both probes with a diameter within the set range—bubbles with diameters larger than the maximum range value were judged as over-ranged and assumed to have a diameter of the maximum range value from which their volume was calculated) and mean diameter index (MDI) (defined as the percentage of mean bubble diameter reduction between the proximal and distal probes) for each intervention.

Patients received standard postoperative care with the addition of two daily (at 12 h intervals) examinations by a trained physician for the presence of hypo- or hyperactive delirium during the first postoperative week. In accordance with our clinic’s protocol, postoperative analgesia was conducted using paracetamol and tramadol. Patients that exhibited signs of hyperactive delirium were sedated using a continuous infusion of dexmedetomidine. The duration of postoperative mechanical ventilation, total volume of drainage, time spent in the ICU, duration of hospital stay and occurrence of postoperative atrial fibrillation (AF) were noted for each patient.

Postoperative delirium was clinically diagnosed using the Confusion Assessment Method for the intensive care unit (CAM-ICU). POD type and severity were evaluated using the Richmond Agitation–Sedation scale (RASS) [15,16].

This study was conducted in accordance with the principles of the Declaration of Helsinki. All patients signed a written informed consent form, and the study was approved by the Institutional Review Board of the University Clinical Centre of Serbia (Belgrade, Serbia) (IRB No. 307/26).

### Statistical Analysis

Numerical data are presented as means with 95% confidence intervals, and categorical variables are presented as absolute numbers with percentages. Differences in clinical and laboratory parameters according to the presence of postoperative delirium were assessed using the Independent Sample *t* test for continuous variables and Chi-square tests for categorical variables. Discrimination performance was tested using sensitivity, specificity and positive and negative predictive values. C statistics, representing the area under the receiver operating characteristic (ROC) curve, was used for an overall assessment of the predictive model. Univariate and multivariate logistic regression analyses were performed to assess predictors of POD. Variables that were significant in the univariate regression analysis were included in the multivariate regression model. The significance level was set at *p* < 0.05. Statistical analysis was performed using the IBM SPSS statistical software (SPSS for Windows, release 25.0, SPSS, Chicago, IL, USA).

## 3. Results

Of the 102 patients who underwent first-time isolated on-pump CABG, 30 (29.4%) had postoperative delirium. Patients with POD were significantly older (67.43 (64.37–70.5) years vs. 62.38 (59.79–64.96) years = 0.013). The baseline characteristics and comorbidities of the study population, according to the presence of POD, are shown in Table 1.

There were no differences in preoperative laboratory parameters (CRP, glucose, blood urea nitrogen (BUN), creatinine, GFR, AST, ALT and LDH) between the patients with and without POD (*p* > 0.05), except for the mean fibrinogen serum level, which was higher in patients who had POD than in patients without POD: 4.27 (3.89–4.66) vs. 3.80 (3.62–3.98) (*p* = 0.014).

The operative and postoperative characteristics of the study population, according to the presence of POD, are presented in Table 2. Patients with POD had a longer ICU stay (*p* = 0.004), longer hospital stay (*p* = 0.015) and higher frequency of AF (*p* = 0.004) than patients without POD.

**Figure 1:**
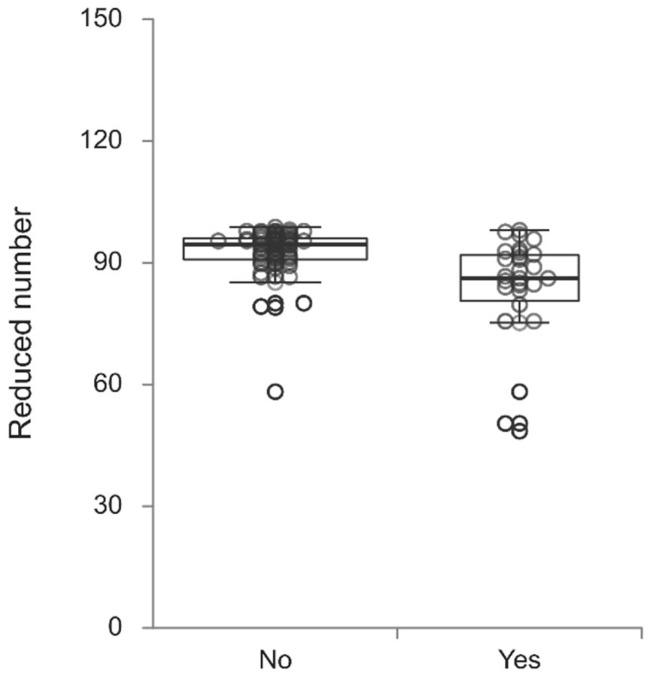
Reduced number (%) of bubbles in patients undergoing coronary artery bypass grafting with postoperative delirium (Yes) compared to patients without postoperative delirium (No) (*p* = 0.001).

**Figure 2:**
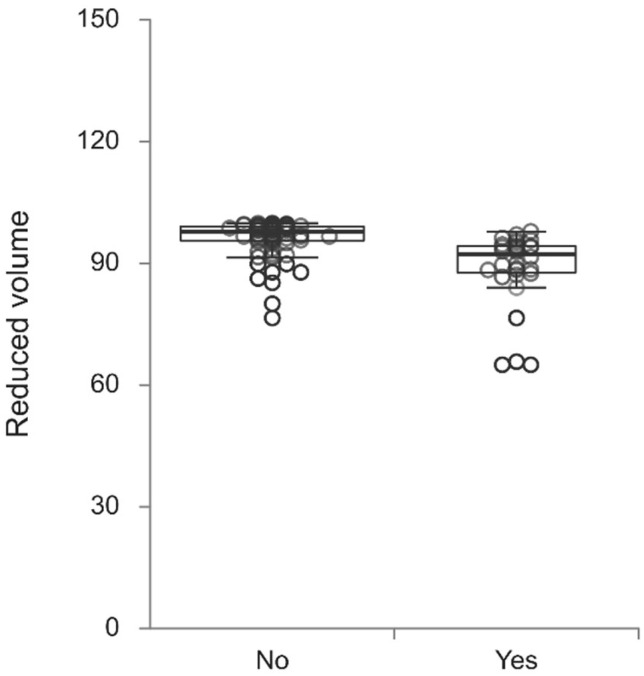
Reduced volume (%) of bubbles in patients undergoing coronary artery bypass grafting with postoperative delirium (Yes) compared to patients without postoperative delirium (No) (*p* < 0.001).

Patients with POD had a higher number of bubbles in the arterial line (*p* < 0.001), higher volume of bubbles in both the venous (*p* = 0.004) and arterial lines (*p* < 0.001) and lower QF values (*p* = 0.039) (Table 3). Lower venoarterial reductions in the number of bubbles (83.0% (77.8–88.1) vs. 92.4% (90.9–93.8), *p* = 0.001) (Figure 1) and volume (88.8% (85.4–92.2) vs. 96.3% (95.2–97.3), *p* < 0.001) (Figure 2) were observed in patients with POD than in those without POD.

**Figure 3:**
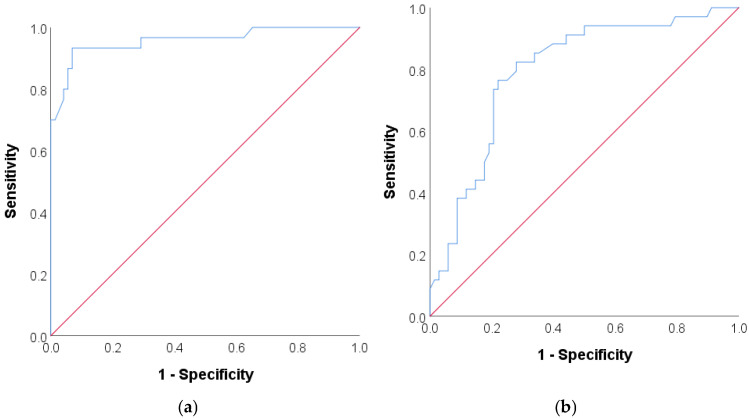
ROC curve of (**a**) arterial volume and (**b**) reduction in volume distinguishing POD.

The ROC curves of arterial volume and reduction in volume values distinguishing POD are presented in Figure 3a,b. Based on the ROC curve analysis, the area under the curve (AUC) values were 0.957 for arterial volume and 0.794 for a reduction in volume. For arterial volume >1 µL, the sensitivity, positive predictive value (PPV), specificity and negative predictive value (NPV) were 76.5%, 86.7%, 94.1% and 88.9%, respectively. For a reduction in volume <95%, the sensitivity, PPV, specificity and NPV were 73.5%, 64.1%, 79.4% and 85.7%, respectively.

Univariate logistic regression analyses revealed that older age (*p* = 0.030), higher preoperative values of fibrinogen (*p* = 0.019) and BUN (*p* = 0.025), the occurrence of AF postoperatively (*p* = 0.005), a higher number of bubbles in the arterial line (*p* < 0.001), higher volumes of bubbles in the arterial and venous lines (*p* = 0.002 and *p* < 0.001, respectively), lower reductions in the number (*p* = 0.001) and volume (*p* < 0.001) of bubbles and lower QF values (*p* = 0.019) were predictors of POD in patients who underwent CABG. In multivariate logistic regression analysis, lower reductions in bubble volume (<95%) and lower arterial volume (>1 µL) were found to be significant independent predictors of POD (*p* = 0.008 and *p* = 0.001, respectively) (Table 4).

## 4. Discussion

This study aimed to investigate the correlation between the number and volume of GME and the onset of POD. The key findings included the following: (a) patients who had a higher number or volume of GME in their arterial line more frequently developed POD; (b) higher reductions in the number and volume of GME during CPB led to the less frequent onset of POD; (c) the independent predictors of the development of POD were arterial GME volume >1 µL and reduction in GME volume <95%.

Previous studies have reported opposing findings on this topic. A study with a design similar to ours was published by Benstoem et al. and showed no correlation between GME load during CPB and worse postoperative outcomes (not even POD), highlighting the improvements in the safety of modern CPB technology in terms of microembolism [17]. The absence of an impact of GME load on POD in this study might be a result of the low POD incidence and small number of patients, as well as the retrospective nature of the study, which might have left some cases of POD unrecognized. Another study conducted in a porcine model provided histopathological evidence of microvascular brain injury related to GME [18]. These histopathological findings are not to be automatically considered clinically significant; nonetheless, they provide firm evidence that GME are capable of inducing brain injury.

Unrelated to CPB, in several studies GME were found to be present in patients with mechanical heart valves years after surgery and are suspected of being responsible for memory impairment [19,20].

A Gampt BCC200 ultrasonic microbubble counter was used to detect the GME in the CPB circuit. [21,22]. Segers et al. found that the Gampt BCC200 overestimates the absolute number and size (and therefore the total volume) of microbubbles, but since the factor of error was fixed and we used the same counter for all our measurements, the results should be comparable even if the absolute numbers might not be accurate [23].

Postoperative Cognitive Decline (POCD) is a widely used outcome for the assessment of postoperative neuropsychiatric events after CABG. It is detected using psychometric tests that have disputable precision, which could be a reason why various previous studies have failed to prove a correlation between CPB and the aforementioned neuropsychiatric decline. Therefore, we believe that delirium might be a more appropriate choice, since its diagnosis is more evident and unequivocal [24,25].

In our study, 29.41% of the patients had POD, which is consistent with previous studies [8]. They were older, had higher preoperative fibrinogen values, spent more time in the ICU and hospital, and more often had postoperative AF. Older age and ICU length of stay are recognized risk factors for POD, as well as EuroScore II [26], which was not found to be correlated with POD in our study. This could be explained by the fact that our study population predominantly had lower EuroScore II values. Fibrinogen, as a coagulation factor and a marker of inflammation, has previously been linked to neuroinflammation and cognitive decline, highlighting the link between neuroinflammation and delirium. CPB evokes an inflammatory response marked by inflammatory mediators such as interleukine-6, interleukine-1β and tumor necrosis factor α, that are associated with blood–brain barrier disruption and neuroinflammation, leading to neuronal damage and cognitive impairment [14,27,28,29,30]. A study conducted by Demertzis et al. found a connection between increased GME load and systemic inflammatory response syndrome, showing that there is reasonable suspicion that GME can induce inflammation and worsen clinical outcome in cardiac surgery patients [31].

As expected, patients who had POD had higher arterial measurements and poorer venoarterial reduction, but they also had higher venous GME volumes. We could argue that this finding implies that the incidence of POD could be lowered by minimizing the introduction of microbubbles (through the two main sources of GME during on-pump surgery: surgical and perfusionist manipulation) and not only by eliminating them once they have entered the CPB circuit. Common perfusionist interventions such as blood sampling or the administration of medications were found to be important sources of GME, emphasizing the importance of simple preventative actions such as de-airing syringes before use. GME load is attenuated by the now-standard use of arterial line filters, lower flow rates, optimal circuit priming technique, the maintenance of normotermia, and the CO_2_ flushing of the circuit. Since the effect of manual de-airing could be insufficient, it is preferable to carry out all surgical manipulations such as aortic (de)cannulation, aortic (de)clamping, heart lifting and defibrillation with utmost attention. Circuit design plays a crucial role in GME prevention, which emphasizes the importance of continuous technological and engineering development [13,32]. In patients who exhibited POD, we found lower QF values (more over-ranged bubbles), proving that in effect they had even higher mean bubble diameter and volume values than those measured.

Multivariate analysis confirmed an independent association between a poor GME volume reduction and POD. This could be because the size of the microbubbles plays a more important role than the number when it comes to disruption of the blood–brain barrier and brain injury [13,33,34].

The present study had several significant limitations. First, the results should be interpreted as hypothesis-generating because of the exploratory nature of the data analysis. This suggests that the findings are preliminary and are intended to guide the quality improvement of local practices and future studies, rather than to provide definitive conclusions. Second, only patients who underwent CABG were included in the study, and further studies are needed to assess the effect of GME on other types of cardiac surgery procedures. Third, considering the size and scope of this study, a subanalysis of the various oxygenator types used was not performed and should be incorporated in separate research. Fourth, not all confounding factors (such as frailty, preoperative benzodiazepine use, postoperative episodes of hypoxia, etc.) for postoperative delirium were taken into consideration.

## 5. Conclusions

The data obtained in this study indicates a strong link between GME load during CPB and the occurrence of POD. Our findings suggest that all feasible measures should be undertaken to prevent gas from being introduced into the CPB circuit, as well as to eliminate GME that have already been generated, before returning blood to the patient. As the consequences of neurological damage mediated by GME may not always be detectable in clinical settings, further studies are required to assess the clinical impact of this event.

## Figures and Tables

**Table 1:** Sociodemographic characteristics and comorbidities of patients undergoing coronary artery bypass grafting with and without postoperative delirium.

Variable	Postoperative Delirium		*p*
	No (*n* = 72)	Yes (*n* = 30)	
Gender, *n* (%)			
Male	58 (80.6)	23 (76.7)	0.658
Female	14 (19.4)	7 (23.3)	
Age, mean (95%CI)	62.38 (59.79–64.96)	67.43 (64.37–70.5)	**0.013**
BMI, mean (95%CI)	27.74 (26.65–28.82)	28.03 (26.62–29.43)	0.760
BSA, mean (95%CI)	2.0 (1.94–2.06)	2.05 (1.97–2.14)	0.377
EuroScore II, mean (95%CI)	1.50 (1.30–1.71)	1.29 (1.12–1.46)	0.199
Anemia, *n* (%)	10 (13.9)	9 (30.0)	0.057
Diabetes mellitus, *n* (%)	30 (41.7)	15 (50.0)	0.440
Preoperative AF, *n* (%)	7 (9.7)	2 (6.7)	0.620

BMI, body mass index; BSA, body surface area; AF, atrial fibrillation.

**Table 2:** Operative and postoperative characteristics of patients undergoing coronary artery bypass grafting with and without postoperative delirium.

Operative and Postoperative Characteristics, Mean (95%CI)	Postoperative Delirium		*p*
	No (*n* = 72)	Yes (*n* = 30)	
CPB (minutes), mean (95%CI)	70.22 (65.3–75.13)	71.58 (61.71–81.45)	0.636
AoCl (minutes), mean (95%CI)	39.20 (35.92–42.47)	39.37 (32.65–46.09)	0.736
pO2 (kPa), mean (95%CI)	27.49 (26.11–28.86)	29.61 (27.22–32.0)	0.251
Cardioplegia (mL), mean (95%CI)	1143.5 (1071.17–1215.79)	1131.58 (1024.94–1238.21)	0.918
Reperfusion (minutes), mean (95%CI)	26.85 (24.34–29.35)	28.84 (24.99–32.70)	0.920
Temperature (°C), mean (95%CI)	33.98 (33.69–34.27)	34.08 (33.58–34.58)	0.391
CS (mL), mean (95%CI)	443.5 (386.8–500.2)	419.74 (356.32–483.15)	0.172
Lactate CPB (mmol/L), mean (95%CI)	2.0 (1.83–2.18)	2.5 (1.91–3.09)	0.248
Glycemia CPB (mmol/L), mean (95%CI)	7.62 (5.41–9.82)	7.19 (6.42–7.97)	0.560
Duration of operation (minutes), mean (95%CI)	256.96 (246.96–266.74)	256.84 (243.02–270.67)	0.413
Duration of intubation (hours), mean (95%CI)	10.33 (9.27–11.38)	9.47 (7.83–11.12)	0.935
Intensive care unit time(days), mean (95%CI)	3.13 (2.70–3.56)	4.21 (3.37–5.05)	**0.004**
Hospital stay (days), mean (95%CI)	6.83 (6.44–7.21)	8.47 (7.29–9.66)	**0.015**
Total drainage (mL), mean (95%CI)	706.63 (630.96–782.30)	638.68 (523.44–753.93)	0.346
Postoperative AF, *n* (%)	17 (28.3)	16 (61.5)	**0.004**

CPB, cardiopulmonary bypass; AoCl, aortic clamp; pO2, partial pressure of oxygen on cardiopulmonary bypass; CS, cell saver; AF, atrial fibrillation.

**Table 3:** Number and volume of bubbles in arterial and venous lines of patients undergoing coronary artery bypass grafting with and without postoperative delirium.

Variable, Mean (95%CI)	Postoperative Delirium		*p*
	No (*n* = 72)	Yes (*n* = 30)	
Venous number, mean (95%CI)	34,707.4 (30,428.1–38,986.7)	36,230.2 (31,442.3–41,018.2)	0.630
Arterial number, mean (95%CI)	2389.4 (2033.9–2745.0)	5382.8 (4127.8–6637.8)	**<0.001**
Venous volume (µL), mean (95%CI)	12.4 (9.7–15.1)	24.2 (16.8–31.6)	**0.004**
Arterial volume (µL), mean (95%CI)	0.29 (0.22–0.36)	1.82 (1.43–2.21)	**<0.001**
QF (%), mean (95%CI)	99.8 (99.6–99.9)	99.1 (98.6–99.7)	**0.039**
MDI (%), mean (95%CI)	30.2 (27.1–33.3)	26.4 (22.9–29.9)	0.103

QF, quality factor; MDI, mean diameter index.

**Table 4:** Univariate and multivariate logistic regression analysis with presence of postoperative delirium as dependent variable.

Variable	Univariate Logistic Regression			Multivariate Logistic Regression		
	*p*-Value	OR	95%CI for OR	*p*-Value	OR	95%CI for OR
Age	**0.030**	1.055	1.005–1.108			
Fibrinogen (preoperative)	**0.019**	1.921	1.115–3.310			
BUN (preoperative)	**0.025**	1.180	1.021–1.365			
AF (postoperative)	**0.005**	4.047	1.535–10.668			
Arterial number	**<0.001**	1.001	1.000–1.001			
Reduction in number	**0.001**	0.890	0.931–0.954			
Venous volume	**0.002**	1.052	1.019–1.087			
Arterial volume >1 µL	**<0.001**	13.750	4.962–38.104	**0.001**	10.777	2.714–42.790
Reduction in volume <95%	**<0.001**	10.714	4.093–28.047	**0.008**	5.392	1.550–18.758
QF	**0.019**	0.389	0.177–0.855			

AF, atrial fibrillation; BUN, blood urea nitrogen; CI, confidence interval; OR, odds ratio; QF, quality factor.

## Data Availability

The datasets that were obtained in this study can be made available by the corresponding author upon reasonable request.
